# Population pharmacokinetics of ritonavir-boosted atazanavir in subsequent-line treatment in African children with HIV

**DOI:** 10.1128/aac.00592-26

**Published:** 2026-07-17

**Authors:** Jennie van Dyk, Catriona Waitt, Henry Mugerwa, Lubbe Wiesner, Catherine Orrell, Alasdair Bamford, Diana M. Gibb, Helen M. McIlleron, Angela Colbers, David M. Burger, Paolo Denti, Roeland E. Wasmann

**Affiliations:** 1 Division of Clinical Pharmacology, Department of Medicine, University of Cape Town, Cape Town, South Africa; 2 Infectious Diseases Institute, Makerere University College of Health Sciences, Kampala, Uganda; 3 Department of Women’s and Children’s Health, University of Liverpool, Liverpool, United Kingdom; 4 Joint Clinical Research Centre 108112 , Lubowa, Uganda; 5 Desmond Tutu HIV Centre, University of Cape Town, Cape Town, South Africa; 6 HIV and Infectious Diseases Unit, South African Medical Research Council 59097, Cape Town, South Africa; 7 Medical Research Council Clinical Trials Unit at University College London 524254, London, England, United Kingdom; 8 Infection, Immunity & Inflammation Department, UCL Great Ormond Street Hospital Institute of Child Health, London, United Kingdom; 9 Wellcome Centre for Infectious Diseases Research in Africa, Institute of Infectious Disease and Molecular Medicine, University of Cape Town, Cape Town, South Africa; 10 Department of Pharmacy, Pharmacology & Toxicology, Radboud University Medical Center 6034, Nijmegen, the Netherlands; Providence Portland Medical Center, Portland, Oregon, USA

**Keywords:** antiretroviral therapy, pediatric, NONMEM, drug interactions

## Abstract

Ritonavir-boosted atazanavir (atazanavir/r) is an effective once-daily option for pediatric subsequent-line antiretroviral therapy when used with two nucleoside reverse transcriptase inhibitors (NRTIs). Tuberculosis co-treatment complicates its use because rifampicin markedly induces atazanavir/r clearance. Although twice-daily atazanavir/r can overcome this interaction in adults, data in children are lacking. We aimed to characterize atazanavir population pharmacokinetics in children with HIV and simulate the effect of rifampicin co-treatment. Atazanavir concentration-time data in African children with HIV from CHAPAS-4 (ISRCTN22964075) and VirTUAL (NCT03923231) were analyzed by nonlinear mixed-effect modeling. We investigated the effect of weight, age, atazanavir formulation, ritonavir dose, and NRTI backbone (tenofovir alafenamide [TAF]-emtricitabine, abacavir-lamivudine, or zidovudine-lamivudine). Simulations were performed across weight bands to evaluate atazanavir/r exposures under standard conditions and, using adult-derived induction effects, predict exposures and possible dosing regimens during rifampicin co-treatment. Seventy children were included, with a median (range) age of 10.9 (3.2–17.7) years and weight of 29 (15–85) kg. A two-compartment model with sequential zero- and first-order absorption best described atazanavir disposition. The estimated typical value of atazanavir clearance was 4.8 L/h for a 27-kg individual. Atazanavir pharmacokinetics in children were unaffected by the NRTI backbone. Once-daily atazanavir/r with current dosing guidelines achieved adequate exposures across weight bands. When simulating pharmacokinetics during rifampicin co-treatment, twice-daily atazanavir/r is expected to restore exposures to levels comparable to once-daily dosing without rifampicin. These findings provide a framework for future clinical evaluation in children with HIV and tuberculosis.

## INTRODUCTION

In 2024, 1.4 million children under 15 years of age were living with HIV globally, with over 85% living in high-tuberculosis burden settings (1, 2). Despite progress in treatment accessibility, coverage in children lags behind that of adults, with an increasing number developing resistance to initial-line antiretroviral therapy (3). As viral load monitoring expands, more children are switching from initial to subsequent-line therapy, and there is an urgent need for safe and effective pediatric regimens (4, 5).

The current World Health Organization (WHO)-preferred initial-line regimen is dolutegravir with an optimized nucleoside reverse transcriptase inhibitor (NRTI) backbone. Dolutegravir-based regimens are also preferred as subsequent-line therapy for people living with HIV, who are failing non-dolutegravir-based regimens. However, in cases where dolutegravir is failing, contraindicated, or not available, boosted protease inhibitors (bPIs) are recommended (6).

bPIs are a highly effective subsequent-line therapy when combined with two NRTIs. Lopinavir is the only available protease inhibitor co-formulated with ritonavir for pediatric use, but it requires twice-daily dosing, has poor palatability, and is less well tolerated compared to other bPIs (7). Ritonavir-boosted darunavir (darunavir/r) can be administered once daily and has a high barrier to resistance, but its use is contraindicated in children younger than 3 years and is relatively costly (7). Ritonavir-boosted atazanavir (atazanavir/r) can be used in children aged 3 months and older, providing an effective, less costly once-daily alternative. If hyperbilirubinemia (observed in approximately 25% of pediatric patients receiving atazanavir/r [6]) does not result in treatment discontinuation, atazanavir/r may be preferred over lopinavir/r in pediatric subsequent-line regimens (8).

The WHO recommends atazanavir/r to be dosed at 200/100 mg (14 to <25 kg) or 300/100 mg (25 to <35 kg) (6). When dosed at 200/75 mg (14 to <25 kg) or 300/100 mg (25 to <35 kg) in children aged 3–15 years, atazanavir/r achieved adult-equivalent exposures with HIV and virological suppression when combined with an optimized NRTI backbone (7, 9).

The CHAPAS-4 trial demonstrated that tenofovir alafenamide (TAF)-emtricitabine resulted in superior virologic suppression over standard-of-care NRTI backbones when used in pediatric subsequent-line regimens (7). TAF can be used at lower doses with a lower risk of renal and bone toxicity compared with tenofovir disoproxil fumarate (TDF) (10). While TDF has been reported to increase atazanavir clearance in children (11), the impact of TAF coadministration on atazanavir pharmacokinetics in children remains uncharacterized.

Rifampicin is a cornerstone in antitubercular treatment and a potent inducer of various drug-metabolizing enzymes (e.g., cytochrome P450) and transporters (e.g., P-glycoprotein), which play important roles in the uptake and metabolism of antiretrovirals (12). Consequently, co-administration of rifampicin can reduce antiretroviral exposure, complicating HIV-tuberculosis co-treatment. In adults, once-daily atazanavir exposure is substantially reduced when co-administered with rifampicin, but switching to twice-daily atazanavir/r restores therapeutic concentrations and is well tolerated (13). Comparable data in children are lacking, requiring further investigation to better inform pediatric dosing strategies.

In this study, we developed a population pharmacokinetic model of atazanavir in children and adolescents with HIV to investigate the effect of NRTI backbones and other covariates on its pharmacokinetics. Using this pediatric model, we also explored dosing strategies for atazanavir/r when administered with rifampicin using adult-derived induction effects in our simulations.

## MATERIALS AND METHODS

### Study design

Data in children and adolescents receiving atazanavir/r were obtained from the CHAPAS-4 (ISRCTN22964075) trial and VirTUAL (NCT03923231) study. Both studies and their nested pharmacokinetic sub-studies were approved by relevant local and national ethics committees. Written informed consent was obtained from parents/caretakers, with assent obtained from children as appropriate, based on age, local country guidelines, and knowledge of HIV diagnosis. All study procedures were conducted in accordance with the Declaration of Helsinki and Good Clinical Practice guidelines.

CHAPAS-4 was an open-label, multicenter, 4 × 2 factorial randomized trial conducted across sites in Zambia, Uganda, and Zimbabwe (7). Eligible participants were children with HIV aged 3–15 years weighing at least 14 kg, receiving initial-line non-nucleoside reverse transcriptase-containing antiretroviral regimen with an abacavir-, zidovudine-, or stavudine-containing NRTI backbone and failing according to current WHO criteria (confirmed viral load >1,000 copies/mL after adherence counseling or CD4 criteria for failure or clinical criteria for failure). Participants were randomized to receive one of four subsequent-line anchor drugs and were also randomized to TAF-based or standard-of-care backbone. Those randomized to receive atazanavir/r were dosed once daily according to WHO weight bands (Table S1). Children weighing 14–19.9 or 20–24.9 kg received 200 mg of atazanavir (using 100 mg atazanavir tablets) and 75 mg of ritonavir if 25-mg tablets were available or a 100-mg ritonavir tablet otherwise. Children weighing 25 kg and above were treated with 300/100 mg atazanavir/r using a fixed-dose combination tablet. Participants were followed-up for 96 weeks, and intensive pharmacokinetic blood samples were collected at week 6 at pre-dose and 1, 2, 4, 6, 8, 12, and 24 h post-dose. Children in the TAF-based backbone arm had an additional blood sample drawn 0.5 h post-dose. On the day of the pharmacokinetic sampling, antiretroviral therapy was administered under direct supervision with food. Any co-medication was administered at least 2 h after the study drugs.

Children receiving treatment for comorbidities with significant drug-drug interactions with study medications were excluded, except for tuberculosis, for which a predefined management strategy was implemented. During rifampicin-based tuberculosis treatment, and for 4 weeks thereafter, temporary modifications to antiretroviral therapy were required to account for drug-drug interactions. Because rifampicin is contraindicated with atazanavir/r, children who developed tuberculosis discontinued atazanavir/r and were re-randomized to receive either twice-daily dolutegravir or double-dose lopinavir/ritonavir for the duration of the tuberculosis management period. As a result, no pharmacokinetic data were collected for the co-administration of atazanavir/r with rifampicin. Participants remained in the trial and followed these protocol-specified adjustments before returning to their originally randomized regimen once rifampicin was discontinued.

The VirTUAL consortium conducted an observational, opportunistic pharmacokinetic study investigating atazanavir/r pharmacokinetics in understudied populations. South African and Ugandan children and adolescents (<18 years) living with HIV and receiving atazanavir-based or subsequent-line antiretroviral therapy with concurrent rifamycin-based tuberculosis treatment were included in the study. Written parental or guardian consent and assent from children aged ≥7 years (South Africa) or aged ≥8 years (Uganda) were required in accordance with national guidelines, with dissent respected at any age. Participants with conditions likely to interfere with study participation or who declined assent were excluded.

Participants were enrolled during routine clinic visits and underwent sparse pharmacokinetic sampling over three visits 1 to 6 months apart. Atazanavir was administered either as 150- or 200-mg tablets with 100-mg ritonavir tablets or as a single 300-/100-mg atazanavir/r fixed-dose combination tablet (Table S1). Dosing and formulations were dependent on local standard of care and varied by country and drug availability. When atazanavir/r was administered in the clinic, dosing was observed and pharmacokinetic samples collected pre-dose and at 2, 4, and 6 h post-dose. If atazanavir/r was self-administered at home, the dosing was reported, and blood samples were collected once on arrival at the clinic and again after completion of routine clinical procedures.

### Assay procedure

Blood samples were immediately centrifuged after collection and stored at −80°C prior to being shipped for analysis. Atazanavir plasma concentrations obtained from CHAPAS-4 were measured at the Department of Pharmacy, Pharmacology & Toxicology, Radboud University Medical Center, Nijmegen, the Netherlands, using a validated high-performance liquid chromatography quantification method with a lower limit of quantification (LLOQ) of 0.105 mg/L for atazanavir. Plasma samples from the VirTUAL study were quantified at the Division of Clinical Pharmacology, University of Cape Town, South Africa, using a validated multiplex high-performance liquid chromatography with tandem mass spectrometry detection assay with an LLOQ of 0.030 mg/L for atazanavir. Both laboratories participate in the same quality assurance programs for external assay validation (14).

### Population pharmacokinetic analysis

Model development was performed using intensive pharmacokinetic data from the CHAPAS-4 trial. Concentration-time data were analyzed using nonlinear mixed-effects modeling in NONMEM v7.5.1 (ICON Development Solutions, Ellicott City, MD, USA) with the first-order conditional estimation with interaction (FOCE-I) parameter estimation. Perl Speaks NONMEM v5.4.0, Pirana v24.9.0, and R v4.4.3 were used for processing and visualization.

One- and two-compartment disposition models were investigated with first-order absorption (with or without sequential lag time and zero-order absorption) and first-order elimination. Between-subject variability was included on clearance, and between-occasion variability was included on absorption parameters, where each dose is considered a separate occasion (15). Combined additive and proportional error models to describe residual unexplained variability were evaluated. Observations that fell below the LLOQ were handled using the M7 +method (16). Participants with atazanavir pre-dose concentrations below the LLOQ or with concentration-time profiles entirely below the LLOQ were considered non-adherent, and these observations were excluded from the analysis. For participants with pre-dose concentrations above the LLOQ but inconsistent with their self-reported doses, we applied the B2 compartment initialization method (17), where the first observed concentration was set as the initial central compartment value and allowing for residual variability.

Allometric scaling was included *a priori* on clearance and volume parameters with fixed exponents of 0.75 and 1, respectively, and we tested using either total body weight or fat-free mass as body size descriptors (18). After accounting for allometry, we performed a stepwise covariate search with a forward inclusion of *P* < 0.05 and backward elimination of *P* < 0.01. We investigated the effects of age, formulation, ritonavir dose, ritonavir exposure, and NRTI backbone on atazanavir disposition.

Model performance was assessed using goodness-of-fit plots and visual predictive checks. Data from the VirTUAL study initially served as an external control to validate the proposed atazanavir model, where we compared model-predicted and observed concentrations using a visual predictive check. Following this evaluation, both data sets were included in joint parameter estimation of the final model. Parameter precision of the final joint model was evaluated using the sampling importance resampling (SIR) procedure (19).

### Simulations

The final model was used to simulate current WHO-recommended once-daily atazanavir doses (6) across WHO-harmonized weight bands (20), collapsed into four groups to reflect the atazanavir/r dosing schedule used in the CHAPAS-4 trial: 15–19.9 kg and 20–24.9 kg (200 mg); 25–34.9 kg and 35–44.9 kg (300 mg). Weight bands were populated *in silico* with 1,000 individuals using a uniform weight distribution, and exposures (AUC_0-24_) and trough concentrations (C_trough_) were simulated.

In addition, we investigated potential dose adjustments in children receiving concurrent rifampicin-based tuberculosis treatment. Rifampicin induction effects were informed by pharmacokinetic findings from a dose-escalation study in Ugandan adults co-treated with atazanavir/r and rifampicin (13) and implemented in our simulations as follows: for once-daily atazanavir/r with rifampicin, atazanavir clearance was increased 3.12-fold, and bioavailability was reduced by 53%. In scenarios where atazanavir/r was administered twice-daily with rifampicin, atazanavir clearance was increased 2.04-fold, and no effect on bioavailability was applied.

Simulated exposures were compared with the geometric mean AUC_0-24_ of adults receiving 300/100 mg atazanavir/r once daily without rifampicin (21). For C_trough_ comparisons, both the minimum effective concentration (MEC, 0.15 mg/L) (22) and protein-adjusted 90% inhibitory concentration (PA-IC_90_, 0.014 mg/L) (23) were used.

## RESULTS

### Study population

We included data from 60 participants (488 concentration-time points) in the CHAPAS-4 trial and 10 participants (30 concentration-time points) in the VirTUAL study. Two participants from VirTUAL and three pre-dose observations in CHAPAS-4 were excluded from analysis for non-adherence. Compartment initialization was applied to one participant from the CHAPAS-4 trial. Of the total 518 observations included, two post-dose concentrations fell below the LLOQ. Ritonavir AUC_0-24_ was available for CHAPAS-4 participants from a previous non-compartmental analysis (24). Four CHAPAS-4 trial participants received 200/100 mg atazanavir/r and were in the lower ([14–19.9 kg] and [20–24.9 kg]) weight bands. A summary of the study population can be found in Table 1.

**TABLE 1:** Population summary of children and adolescents receiving atazanavir/ritonavir in the CHAPAS-4 and VirTUAL studies^*c*^

Characteristic	Study		
	Overall	CHAPAS-4	VirTUAL
	*N* = 70^*a*^	*N* = 60^*a*^	*N* = 10^*a*^
Age (years)	10.9 (3.2, 17.7)	9.9 (3.2, 15.6)	15.0 (12.0, 17.7)
Sex (male)	39 (56%)	32 (53%)	7 (70%)
Height (cm)	133 (102, 168)	129 (102, 162)	158 (132, 168)
Weight (kg)	29 (15, 85)	26 (15, 64)	46 (32, 85)
Weight bands (kg)			
14–19.9	12 (17%)	12 (20%)	NA
20–24.9	16 (23%)	16 (27%)	NA
25–34.9	20 (29%)	18 (30%)	2 (20%)
≥35	22 (31%)	14 (23%)	8 (80%)
Atazanavir/ritonavir dose (mg)			
150/100	2 (2.9%)	NA	2 (20%)
200/75	24 (34%)	24 (40%)	NA
200/100	5 (7.1%)	4 (6.7%)	1 (10%)
300/100^*b*^	39 (56%)	32 (53%)	7 (70%)
Ritonavir AUC_0-24_ (mg.h/L)	16.3 (11.7, 24.2)	16.3 (11.7, 24.2)	NA
NRTI backbone			
TAF/FTC	34 (49%)	34 (57%)	NA
TDF/3TC	5 (7.1%)	NA	5 (50%)
ABC/3TC	22 (31%)	17 (28%)	5 (50%)
ZDV/3TC	9 (13%)	9 (15%)	NA

^*a*^ Median (min, max); *N* (%).

^*b*^ Fixed-dose combination.

^*c*^ TAF/FTC, tenofovir alafenamide/emtricitabine; TDF/3TC, tenofovir disoproxil/lamivudine; ABC/3TC, abacavir/lamivudine; ZDV/3TC, zidovudine/lamivudine; NA, not applicable.

### Pharmacokinetic analysis

A two-compartment model with lag time and sequential zero- and first-order absorption with first-order elimination best described atazanavir disposition. Allometric scaling using total body weight improved model fit (change in objective function [dOFV] =−170.7) and was chosen over fat-free mass for practicality and ease of comparison with other studies as both covariates performed similarly (dOFV = 1.885, compared to model with fat-free mass allometric scaling). After accounting for body size, age was not a significant covariate in the model. The estimated typical value of atazanavir clearance was 4.8 L/h for a 27-kg individual.

To account for high variability in pre-dose concentrations, a scaling factor was applied (and later fixed for model stability) as a fixed effect to inflate the between-occasion variability on bioavailability (dOFV = −12.30; *P* < 0.001) for the concentrations measured after an unobserved dose.

Participants receiving atazanavir/r as separate atazanavir and ritonavir tablets (200/75 mg or 200/100 mg atazanavir/r) had 26.7% (95% CI: 13.1%–35.0%) lower atazanavir bioavailability compared with those receiving the 300/100 mg atazanavir/r fixed-dose combination (dOFV = –33.89; *P* < 0.001). The effect of ritonavir AUC was initially included as a power model on atazanavir clearance (dOFV = −6.268; *P* < 0.02) with an inverse relationship but was not retained in the model after backward elimination of covariates. No significant effect of the backbones TAF-emtricitabine, abacavir-lamivudine, or zidovudine-lamivudine on atazanavir pharmacokinetics was identified.

External validation, in which the CHAPAS-4-derived model was used to predict atazanavir concentrations in the VirTUAL data set, demonstrated adequate predictive performance, Fig. S1. Following validation, parameters were re-estimated using the combined CHAPAS-4 and VirTUAL data sets. Study-specific differences were tested in the model, including in the residual error, but no significant difference was found. We retested the effect of atazanavir tablet formulation on bioavailability using the VirTUAL data set, which resulted in a greater reduction in objective function value (dOFV = −5.7) than a ritonavir dose effect and was therefore retained in the final model.

**Fig 1:**
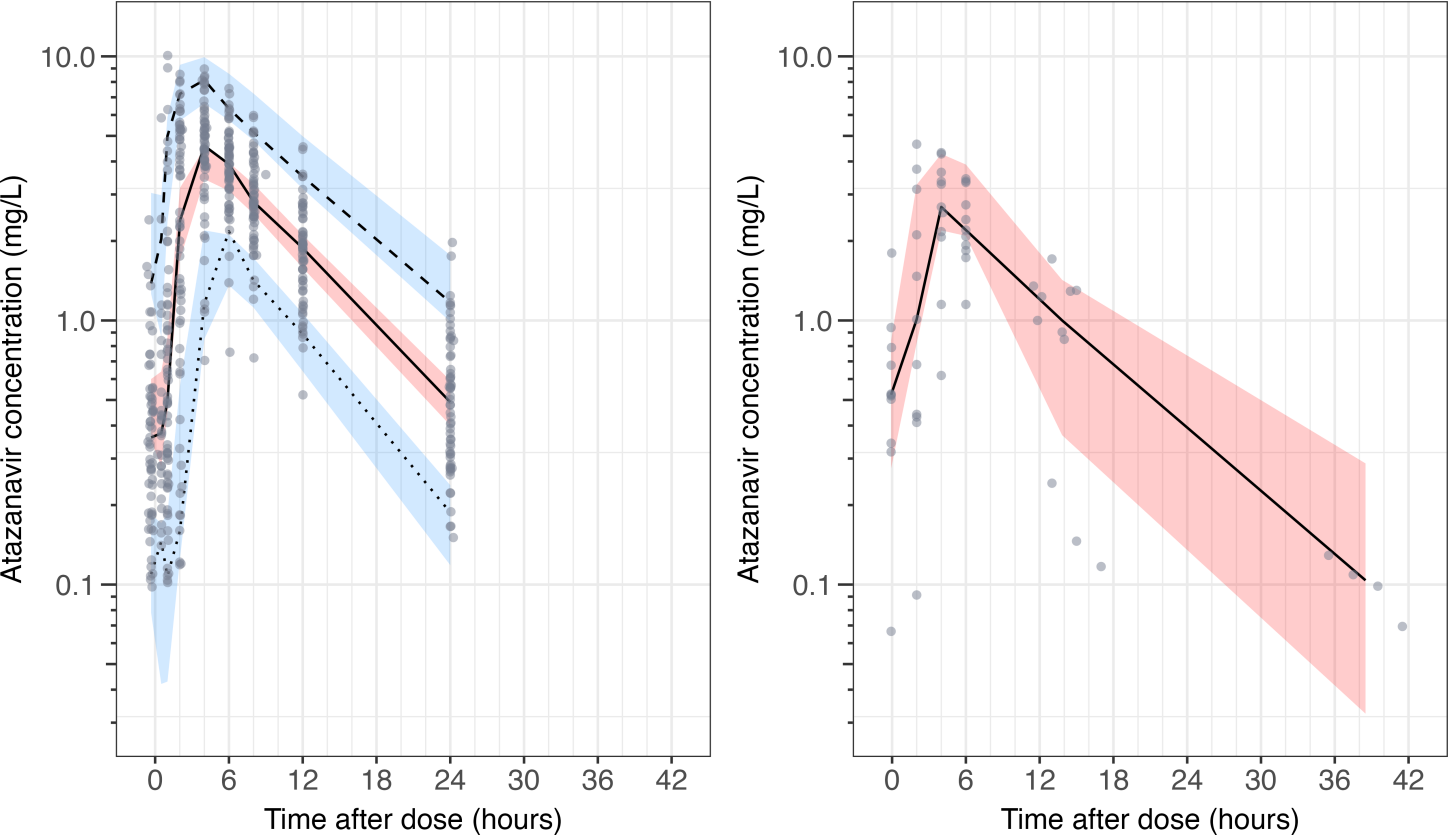
Visual predictive check for the final atazanavir model in children from the CHAPAS-4 (left) and VirTUAL (right) studies. Solid circles represent observed data. The solid, dotted, and dashed lines represent the 50th, 5th, and 95th percentiles of observed data, respectively. The shaded areas represent the model-predicted 95% confidence intervals for the same percentiles. For the VirTUAL study (*n* = 10), only the observed and simulated median (50th percentile) are shown as the sample size was insufficient to plot the 5th and 95th percentiles.

Final parameter estimates and an adequate model fit are provided in Table 2 and Fig. 1, respectively.

**TABLE 2:** Population pharmacokinetic model parameter estimates^*h*^

Parameter	Atazanavir	
	Typical value	95% confidence interval^*b*^
Clearance, CL (L/h)^*a*^	4.8	4.37–5.41
Change in CL due to rifampicin (OD ATV/r dose) (-fold)^*d*^	3.12	FIX
Change in CL due to rifampicin (BD ATV/r dose) (-fold)^*d*^	2.04	FIX
Central volume, Vc (L)^*a*^	41.2	37.8–46.0
Lag time (h)^*g*^	1	FIX
Zero-order input, D1 (h)	1.04	0.829–1.28
First-order input, ka (h^−1^)	2.23	1.62–2.73
Bioavailability, F (.)	1	FIX
Change in F for cohorts receiving non-FDC (%)^*c*^	−26.7	−35.0 to −13.1
Change in F due to rifampicin (OD ATV/r dose) (%)^*d*^	−53.0	FIX
Scaling factor on BOV for the unobserved dose (-fold change)	1.43	FIX
Peripheral volume, Vp/F (L)^*a*^	47.5	18.9–113
Intercompartmental clearance, Q/F (L/h)^*a*^	0.418	0.336–0.680
Proportional error (%)	11.8	10.9–13.3
Additive error (mg/L)^*e*^	20% LLOQ	FIX
Between-subject variability, BSV (%CV)^*f*^		
Clearance, CL	18.9	14.5–22.4
Between-occasion variability, BOV (%CV)^*f*^		
Lag time^*g*^	60.5	FIX
Zero-order input, D1	111	67.0–172
First-order input, ka	162	102–225
Bioavailability, F	34.7	30.5–41.8

^*a*^ Allometrically scaled for a 27-kg individual.

^*b*^ Parameter uncertainty was determined by sample importance resampling (SIR).

^*c*^ Includes children <25 kg receiving 2 × 100 mg atazanavir and 4 × 25 mg or 100 mg ritonavir tablets and ≥25 kg participants receiving 200 or 100 mg atazanavir with 100 mg ritonavir tablets.

^*d*^ Rifampicin assumptions extracted from the Kengo et al. model (13). Included for simulations of ATV/r dosing scenarios in children receiving rifampicin.

^*e*^ Additive error fixed to 20% LLOQ. CHAPAS-4 LLOQ, 0.105 mg/L. VirTUAL LLOQ, 0.0300 mg/L.

^*f*^ Pharmacokinetic parameter variability is reported here as the percent coefficient of variation (%CV) calculated as follows: %CV=SQRT[(EXP(ω^2 )-1)]∙100.

^*g*^ The typical value of lag time and its variability were initially estimated and later fixed in the final model for stability.

^*h*^ OD, once-daily; BD, twice-daily; ATV/r, atazanavir/ritonavir; Non-FDC, non-fixed dose combination ATV/r tablets; LLOQ, lower limit of quantification; BSV, between-subject variability; BOV, between-occasion variability.

### Simulations

**Fig 2:**
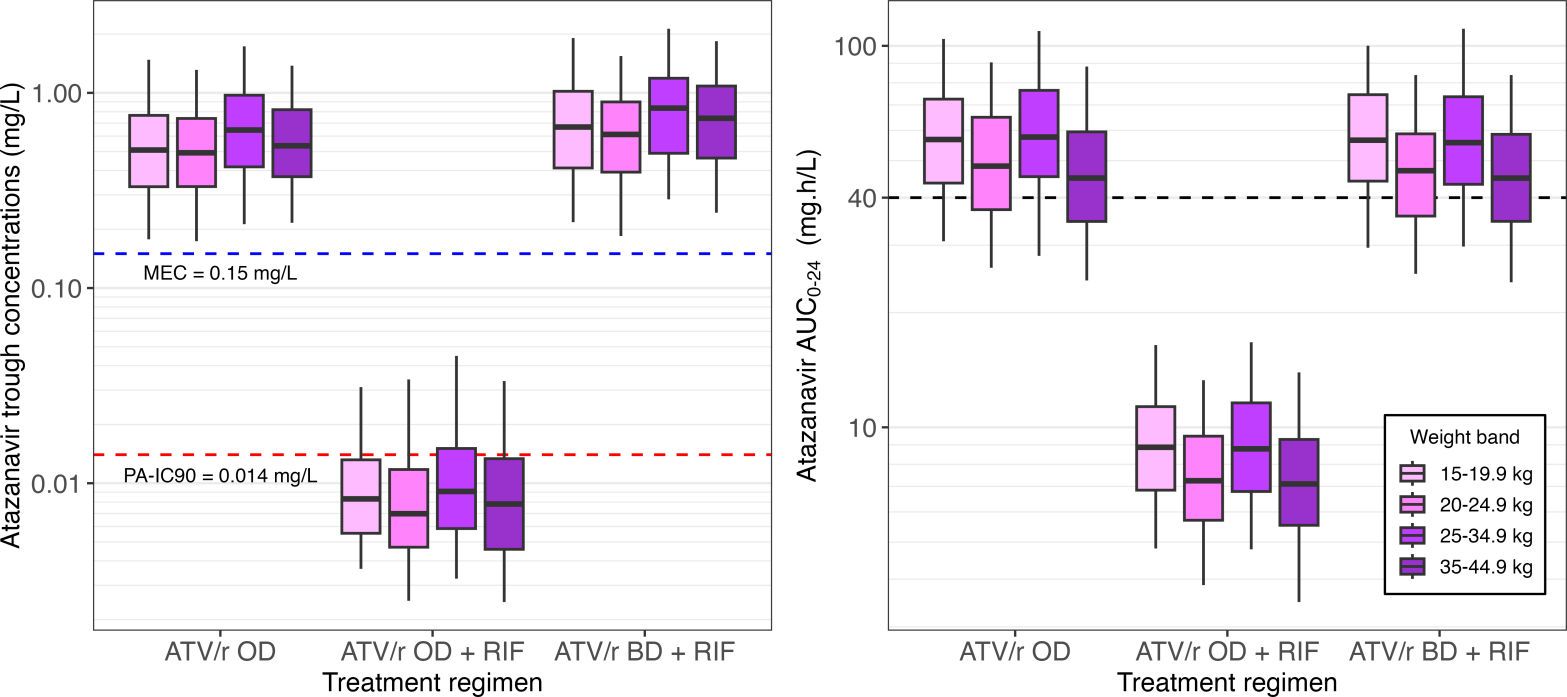
Simulated trough concentrations (left) and atazanavir steady-state AUC_0-24_ (right) in children receiving CHAPAS-4 atazanavir/r doses (Table 1) with or without rifampicin. Boxplots represent the 5th, 25th, 50th, 75th, and 95th data percentiles. The black dashed line represents the geometric mean AUC_0-24_ in adults receiving 300/100 mg atazanavir/r once daily without rifampicin (21). ATV/r, ritonavir-boosted atazanavir. OD, once daily. BD, twice daily. RIF, rifampicin. MEC, minimum effective concentration (22). PA-IC_90_, protein-adjusted 90% inhibitory concentration (23).

The results of the simulations are shown in Fig. 2. Three dosing scenarios were evaluated: once-daily atazanavir/r without rifampicin, once-daily atazanavir/r with rifampicin, and twice-daily atazanavir/r with rifampicin.

Without rifampicin, once-daily atazanavir/r achieved adequate exposures across all weight bands, with 100% and 97.4% of C_trough_ values exceeding the PA-IC_90_ (0.014 mg/L) and MEC (0.15 mg/L), respectively. Rifampicin coadministration is predicted to markedly reduce atazanavir exposure; all simulated C_trough_ values fell below the MEC and over 75% fell below the PA-IC_90_ with once-daily dosing. Twice-daily atazanavir/r is predicted to restore exposures to levels comparable to once-daily dosing without rifampicin, with 100% and 98.6% of simulated C_trough_ values exceeding the PA-IC_90_ and MEC across all weight bands.

## DISCUSSION

We developed a nonlinear mixed-effects model describing the pharmacokinetics of ritonavir-boosted atazanavir in 3- to 17-year-old African children and adolescents receiving subsequent-line antiretroviral therapy. The model structure aligns with a published atazanavir model in adults with HIV receiving 300/100 mg atazanavir/r (13) and was externally validated against concentration-time data from African children and adolescents in the VirTUAL study. The model characterized the effect of body weight and confirmed the appropriateness of the current WHO dosing guidelines, including the new harmonized weight bands (20). Assuming a rifampicin induction effect similar to that recently reported in adults (13), twice-daily atazanavir/r dosing with rifampicin could compensate for the interaction and achieve appropriate atazanavir exposure (AUC_0-24_) and trough concentrations (C_trough_).

Previous studies have reported an effect of TDF-containing backbones on atazanavir pharmacokinetics. In children receiving atazanavir/r, TDF was associated with an approximately 25% increase in apparent atazanavir clearance (11). In contrast, adult data have been inconsistent, and a more recent study of atazanavir/r in adults with HIV reported no effect of TDF on atazanavir clearance or bioavailability (13). The pharmacokinetics of the newer tenofovir prodrug TAF have been investigated in healthy adults receiving atazanavir/r, where coadministration increased TAF and tenofovir exposure without affecting atazanavir pharmacokinetics (25).

In the present analysis, no effect of TAF-emtricitabine, abacavir-lamivudine, or zidovudine-lamivudine on atazanavir/r pharmacokinetics was identified, with comparable atazanavir exposures observed across NRTI backbones (9). This is important as, in the main CHAPAS-4 trial, TAF-emtricitabine demonstrated favorable efficacy, tolerability, low cost, and once-daily dosing convenience (7). Although atazanavir/r produced higher TAF exposure compared with other bPIs, tenofovir concentrations (the primary driver of toxicity [10]) remained well below predefined pediatric toxicity thresholds (26). The effect of atazanavir/r on TAF is, therefore, unlikely to be clinically relevant, and our analysis confirms that TAF does not meaningfully affect atazanavir pharmacokinetics. Taken together, these findings support the use of atazanavir/r with TAF-emtricitabine in subsequent-line therapy for children and adolescents.

A previous non-compartmental analysis of the CHAPAS-4 atazanavir/r pharmacokinetic data identified lower atazanavir exposure in children in the 14–24.9 kg weight band compared to those in the 25–34.9 kg weight band but did not see a similar effect for ritonavir exposure (9). The current analysis attributes this difference to a formulation effect of the atazanavir tablets, whereby standalone atazanavir and ritonavir formulations given to lower weight bands resulted in 26.7% lower atazanavir bioavailability compared to the fixed-dose combination tablet administered to higher weight bands. This may partly reflect adherence challenges associated with the higher pill burden of separate tablets as switching to fixed-dose combinations has been associated with improved adherence in other multi-tablet antiretroviral regimens (27).

We also investigated whether the difference in atazanavir exposure reflects variation in ritonavir dose or exposure, but the model consistently directed to an effect of tablet formulation. Ritonavir doses of 50–100 mg are reported to achieve near-maximal inhibition of bPI clearance (28, 29), suggesting that difference in CYP3A4 inhibition between the 75-mg and 100-mg doses in this study is unlikely to be meaningful. Moreover, ritonavir bioavailability and exposure were consistently higher in participants receiving atazanavir/r regardless of the formulation, and exposures were balanced across weight bands despite lower weight bands receiving standalone ritonavir (30). Differences in ritonavir exposure are, therefore, unlikely to account for the atazanavir bioavailability effect observed in our analysis. Importantly, in the current study, all atazanavir C_trough_ were above the PA-IC_90_ efficacy threshold (9), and atazanavir/r effectively reduced viral load after 96 weeks of treatment (7).

Options for concurrent HIV and tuberculosis therapy in children are limited. Darunavir/r use is limited to children over 3 years old, and twice-daily dosing with rifampicin has been explored in adults but produced an unacceptable risk of hepatotoxicity, limiting its use in HIV and tuberculosis co-treatment (31). Lopinavir/r remains the only recommended bPI option with rifampicin-based therapy for dolutegravir-experienced children (32), but its use requires super-boosting with additional ritonavir, which is poorly tolerated and difficult for caregivers to implement (33).

Guidelines do not currently recommend twice-daily atazanavir/r with rifampicin-based tuberculosis treatment due to the potent induction of CYP3A metabolism (12, 32). Recent adult pharmacokinetic studies suggest this approach may overcome the rifampicin-mediated drug-drug interaction, maintaining therapeutic exposures with generally mild and transient liver enzyme elevations (13, 21). Similar trials have not been conducted in children; hence, we used these adult induction values to simulate dosing scenarios of atazanavir/r with rifampicin. Our simulation results are similar to those of a previous physiologically based pharmacokinetic analysis, which predicted that 300/100 mg atazanavir/r dosed twice daily could achieve adequate trough concentrations in children aged 7 to 18 years old weighing ≥25 kg receiving rifampicin at 10 mg/kg (34). Given that atazanavir/r is approved for use in children, these findings, together with our simulation results, suggest that twice-daily atazanavir/r may be a viable subsequent-line antiretroviral therapy option with rifampicin-based tuberculosis therapy. Prior research has shown that the order of drug introduction and disease state is important for regimen tolerability (21, 35, 36); therefore, clinical studies are needed to confirm and evaluate the safety and efficacy of these findings.

Our study has some limitations. Children receiving atazanavir/r as separate tablets also received different ritonavir doses (75 mg or 100 mg), and the small number boosted with 100 mg may have limited differentiation of ritonavir dose effects from formulation effects in lower weight bands. Regardless of this effect, these doses and formulations produced satisfactory atazanavir concentrations above the efficacy threshold. Although we tested for differences between data from the two laboratories and found no evidence of study-related effects, combining data sets from different sites could still introduce subtle variability or misspecification; however, parameter estimates changed little when re-estimated with the VirTUAL study data, suggesting that any such differences are likely minor. Rifampicin interactions were simulated using adult induction values, and it is unclear if these would differ in children, particularly at younger age groups with immature metabolic enzymes and transporter systems (37). Even in older children, rifampicin induction effects on other antiretrovirals have been shown to differ from those in adults (38–40). Studying the pharmacokinetics and efficacy of twice-daily atazanavir/r with rifampicin within CHAPAS-4 would have been informative, but the small number of eligible participants precluded this within a randomized trial. Nevertheless, our results support this strategy in future trials evaluating atazanavir/r as a subsequent-line antiretroviral therapy option in children receiving concomitant HIV and tuberculosis therapy.

In summary, we characterized ritonavir-boosted atazanavir pharmacokinetics in children with HIV receiving subsequent-line regimens as part of the CHAPAS-4 and VirTUAL studies. Atazanavir pharmacokinetics was consistent for all studied NRTI backbone combinations and supports its use with TAF-emtricitabine for subsequent-line regimens. Assuming rifampicin has a similar induction effect in children as in adults, twice-daily atazanavir/r could restore exposures in children receiving rifampicin-based tuberculosis therapy. Clinical studies are needed to confirm safety and efficacy, and these findings provide a reference to support future investigations of this strategy.

## SUPPLEMENTAL MATERIAL

Supplemental material aac.00592-26-s0001.pdf Table S1; Fig. S1.

ASM does not own the copyrights to Supplemental Material that may be linked to, or accessed through, an article. The authors have granted ASM a non-exclusive, world-wide license to publish the Supplemental Material files. Please contact the corresponding author directly for reuse.
