# Supplementary material for: Population pharmacokinetics of ritonavir-boosted atazanavir in subsequent-line treatment in African children with HIV
Source: Antimicrob Agents Chemother. 2026 Jul 17;70(8):e00592-26. doi: 10.1128/aac.00592-26 (PMC13436004; doi:10.1128/aac.00592-26)
Supplement: Supplementary file 1 [file aac.00592-26-s0001.pdf]

Title: Population pharmacokinetics of ritonavir-boosted atazanavir in subsequent-line treatment in African children with HIV

Corresponding author: Jennie van Dyk

Division of Clinical Pharmacology, Department of Medicine, University of Cape Town, Cape Town, South Africa.

E-mail: vdyjen001@myuct.ac.za

Tel: (+27) 79 691 9945

Table S1. Atazanavir/r doses used in the CHAPAS-4 and VirTUAL studies

Study	Weight band (kg)	100 mg ATV	150 mg ATV	200 mg ATV	25 mg RTV	100 mg RTV	300/100 mg ATV/r	Daily dose (mg)
CHAPAS-4	14–19.9	2	–	–	3	–	–	200/75[a]
	20–24.9	2	–	–	3	–	–	200/75[a]
	25–34.9	–	–	–	–	–	1	300/100
	≥35	–	–	–	–	–	1	300/100
VirTUAL	≥25	–	1	–	–	1	–	150/100
	≥25	–	–	1	–	1	–	200/100
	≥25	–	–	–	–	–	1	300/100

[a] If 25 mg ritonavir was not available, 100 mg ritonavir was used to boost atazanavir for the 14–19.9 kg and 20–24.9 kg weight bands.
ATV, atazanavir; RTV, ritonavir; ATV/r, fixed-dose combination ritonavir-boosted atazanavir.

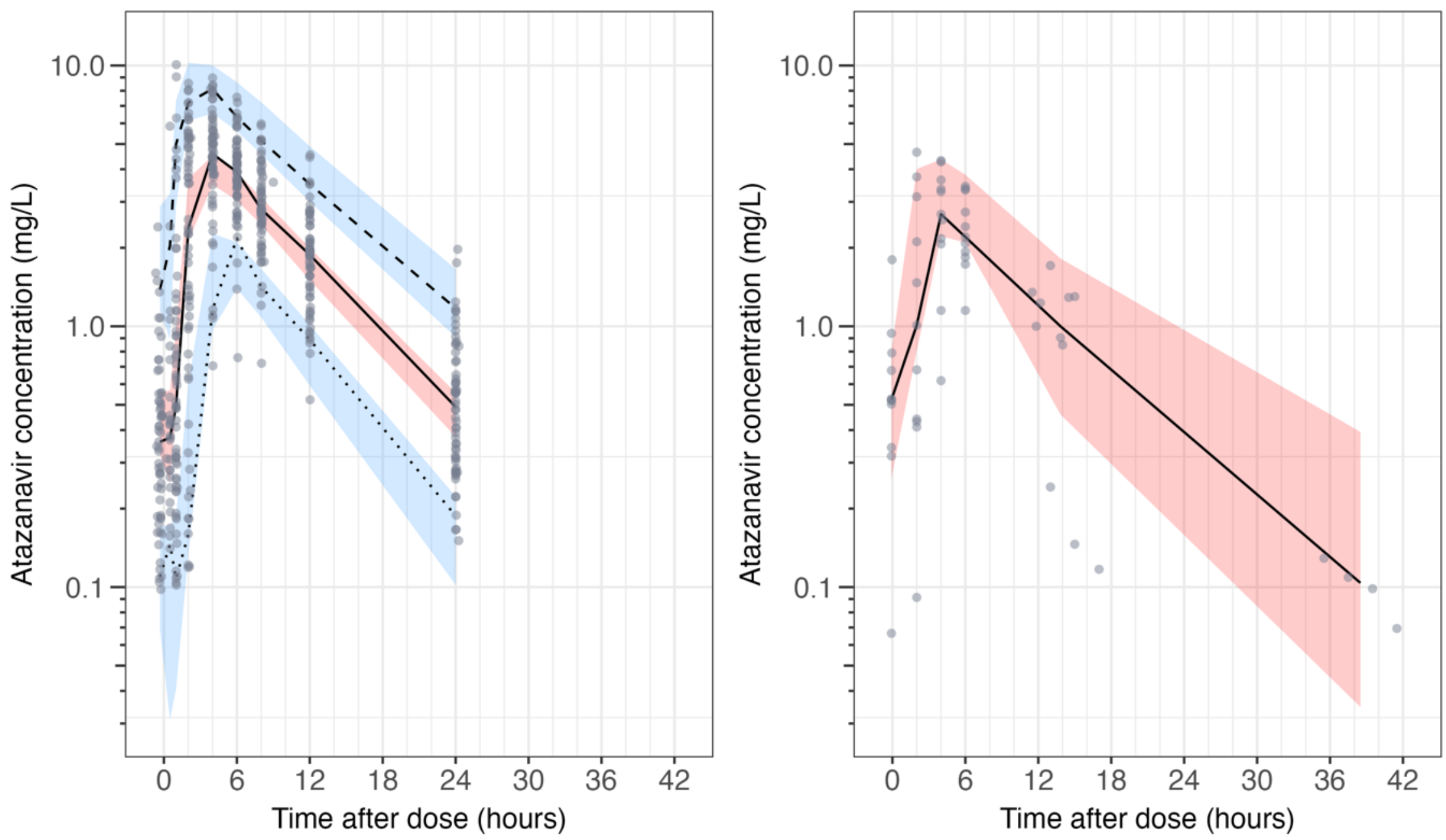

Figure S1. Visual predictive check for the external validation of the atazanavir model. The model developed using CHAPAS-4 data (left) was used to predict concentrations in children from the VirTUAL study (right) without re-estimation. Solid circles represent observed data. The solid, dotted, and dashed lines represent the 50th, 5th, and 95th percentiles of observed data. The shaded areas represent the model-predicted 95% confidence intervals for the same percentiles. For the VirTUAL study (n = 10), only the observed and simulated median (50th percentile) are shown, as the sample size was insufficient to plot the 5th and 95th percentiles.
